# CBAP regulates the function of Akt-associated TSC protein complexes to modulate mTORC1 signaling

**DOI:** 10.1016/j.jbc.2023.105455

**Published:** 2023-11-08

**Authors:** Wei-Ting Liao, Yun-Jung Chiang, Hsin-Fang Yang-Yen, Li-Chung Hsu, Zee-Fen Chang, Jeffrey J.Y. Yen

**Affiliations:** 1 Institute of Molecular Medicine, College of Medicine, National Taiwan University, Taipei, Taiwan; 2 Institute of Biomedical Sciences, Academia Sinica, Taipei, Taiwan; 3 Institute of Molecular Biology, Academia Sinica, Taipei, Taiwan

**Keywords:** Akt, Rheb, small GTPase, cell growth, mTORC1 activation, tumor cell biology

## Abstract

The Akt-Rheb-mTORC1 pathway plays a crucial role in regulating cell growth, but the mechanisms underlying the activation of Rheb-mTORC1 by Akt remain unclear. In our previous study, we found that CBAP was highly expressed in human T-ALL cells and primary tumors, and its deficiency led to reduced phosphorylation of TSC2/S6K1 signaling proteins as well as impaired cell proliferation and leukemogenicity. We also demonstrated that CBAP was required for Akt-mediated TSC2 phosphorylation *in vitro*. In response to insulin, CBAP was also necessary for the phosphorylation of TSC2/S6K1 and the dissociation of TSC2 from the lysosomal membrane. Here we report that CBAP interacts with AKT and TSC2, and knockout of CBAP or serum starvation leads to an increase in TSC1 in the Akt/TSC2 immunoprecipitation complexes. Lysosomal-anchored CBAP was found to override serum starvation and promote S6K1 and 4EBP1 phosphorylation and c-Myc expression in a TSC2-dependent manner. Additionally, recombinant CBAP inhibited the GAP activity of TSC2 complexes *in vitro*, leading to increased Rheb-GTP loading, likely due to the competition between TSC1 and CBAP for binding to the HBD domain of TSC2. Overexpression of the N26 region of CBAP, which is crucial for binding to TSC2, resulted in a decrease in mTORC1 signaling and an increase in TSC1 association with the TSC2/AKT complex, ultimately leading to increased GAP activity toward Rheb and impaired cell proliferation. Thus, we propose that CBAP can modulate the stability of TSC1-TSC2 as well as promote the translocation of TSC1/TSC2 complexes away from lysosomes to regulate Rheb-mTORC1 signaling.

The Tuberous Sclerosis Complex (TSC) protein complex (TSCC) is composed of three proteins, TSC1 (also known as hamartin), TSC2 (also known as tuberin), and TBC1D7 (1, 2), and plays a crucial role in transducing signals from growth factor receptors and oncogenes (3, 4). Mutations in either the *TSC1* or *TSC2* gene can result in the formation of benign tumors, such as renal angiomyolipomas, cardiac rhabdomyomas, and subependymal giant cell astrocytomas, highlighting the importance of TSCC in controlling cellular proliferation (5). The TSCC has a primary biochemical function as a GTPase-activating protein (GAP) toward the small G protein Rheb (Ras homolog enriched in the brain), which maintains Rheb in an inactive GDP-bound state and suppresses mTORC1 activation and cell growth deregulation (6, 7, 8, 9). Nonsense or certain missense mutations in either *TSC1* or *TSC2* impair the GAP activity of TSCC (10), leading to a failure to promote GTP hydrolysis of Rheb molecules and accumulation of active GTP-bound Rheb. The accumulation of active GTP-bound Rheb induces hyperactivation of mTORC1 complexes on the lysosome surface, which is believed to be the primary driver of tumorigenesis in TSC.

The mTORC1 signaling pathway acts downstream of TSCC and is critical in transmitting growth signals, including nutrient and growth factor stimulation, to regulate the balance between anabolism and catabolism (11). Upon activation, mTORC1 promotes protein synthesis by phosphorylating two key effectors, p70S6K and eIF4 binding protein (4EBP1), which subsequently enhance mRNA translation initiation and 5′ cap-dependent mRNA translation, respectively (11, 12). The growth suppressive function of TSCC can be regulated by the upstream phosphotidylinositol-3-kinase (PI3K)/Akt pathway, particularly when cell proliferation is stimulated by growth factors or oncogenic signals. Genetic evidence supports this notion, including the identification of constitutively active oncogenic forms of Akt in murine oncogenic viruses (13) and the presence of inactivating mutations in PTEN, a phosphatase that opposes the action of PI3K, in the majority of human tumors (14, 15). Further studies have shown that TSC2 is a direct substrate of Akt, and Akt-dependent phosphorylation of TSC2 is essential for the activation of the mTORC1/S6K/4EBP1 pathway (16, 17, 18, 19). This supports the notion that both the PI3K/Akt and the TSCC/Rheb/mTORC1 axes are part of one crucial signaling pathway in cell growth and human tumor formation.

Despite the importance of Akt in relieving TSCC-dependent Rheb/mTORC1 signal suppression (16, 17, 18, 19), progress in understanding the underlying mechanism of Akt-mediated Rheb/mTORC1 activation has been relatively slow. While early reports proposed that Akt-mediated phosphorylation destabilizes the TSC1-TSC2 complex, leading to dissociation of TSC1 from TSC2 and subsequent activation of mTORC1 signaling (16, 18), more recent studies have suggested that there was no destabilization of endogenous TSC1 and TSC2 protein complexes before or after TSC2 phosphorylation. Instead, Cai *et al.* (19) observed changes in the membrane partition of TSC2 proteins induced by serum and Akt, while Menon *et al.* (3) reported insulin-induced dissociation of TSC1-TSC2 complexes from the lysosomal membrane. According to this model, the intrinsic GAP activity of TSC complexes remains unchanged, and instead, Rheb-GTP levels are increased due to dissociation from its GAP protein. However, it remains unclear whether PI3K/Akt signaling can directly control the intrinsic GAP activity of the TSC complexes.

T-cell lymphoblastic leukemia (T-ALL) is a highly aggressive subtype of leukemia affecting both pediatric and adult patients (20). Despite advances in treatment, induction failure and early relapse remain common, presenting a significant therapeutic challenge (21). The PI3K-Akt signaling axis has been identified as one of key pathways in T-cell leukemia, with frequent upregulation observed in T-ALL patients and activation of this pathway being associated with poor prognosis, therapeutic resistance, and disease relapse (22, 23, 24, 25). Studies utilizing gene knockout and pharmacologic inhibition have demonstrated that the Rheb/mTORC1/S6K/c-myc pathway plays a crucial role in T-ALL tumor growth, promoting tumor cell survival and proliferation (26, 27).

Previously, we identified CBAP as an interacting protein of the GM-CSF/IL-3/IL-5 receptor common β-chain, which participates in cytokine deprivation-induced apoptosis (28). CBAP is a member of the Mab21 subfamily within the nucleotide transferase protein fold superfamily (29). We have shown that CBAP participates in multiple signal transduction pathways, including chemokine-enhanced T-cell migration and adhesion (30) and T-cell receptor engagement-induced phosphorylation of ZAP-70 and PLCγ1 (31). In 2019, we reported that CBAP proteins are highly expressed in T-cell leukemia tumor tissues and established cell lines, and knockout or knockdown of the CBAP gene suppressed Jurkat cell growth and leukemogenesis in mice (32). The lack of CBAP diminished both the Raf-MEK-ERK and the Akt-mTORC1 signaling pathways in Jurkat cells. Specifically, CBAP was required for Akt-dependent TSC2 phosphorylation at T1462 and S939 in cell-based assays and at T1462 in *in vitro* kinase assays. Finally, confocal imaging analyses revealed that CBAP deficiency resulted in a decrease in the dissociation of TSC2 from lysosomal membranes, consistent with the reduction of mTORC1 signaling when insulin stimulates HeLa cells (32).

Here, we have investigated the mechanistic role of CBAP in the activation of the Akt-regulated Rheb/mTOR pathway. We explored the possibility of protein–protein interaction in modulating the function of TSC1-TSC2 complexes and Rheb/mTORC1 signaling. Our current results strongly suggest that Akt/CBAP can regulate Rheb/mTOR activation by not only promoting the translocation of TSC complexes away from lysosomes but also actively dissociating TSC1 from Akt/TSC2 complexes, thereby decreasing the GAP activity of TSC2 toward Rheb.

## Results

### CBAP influences the stability of TSC1–TSC2 binding in the Akt complex

**Figure 1:**
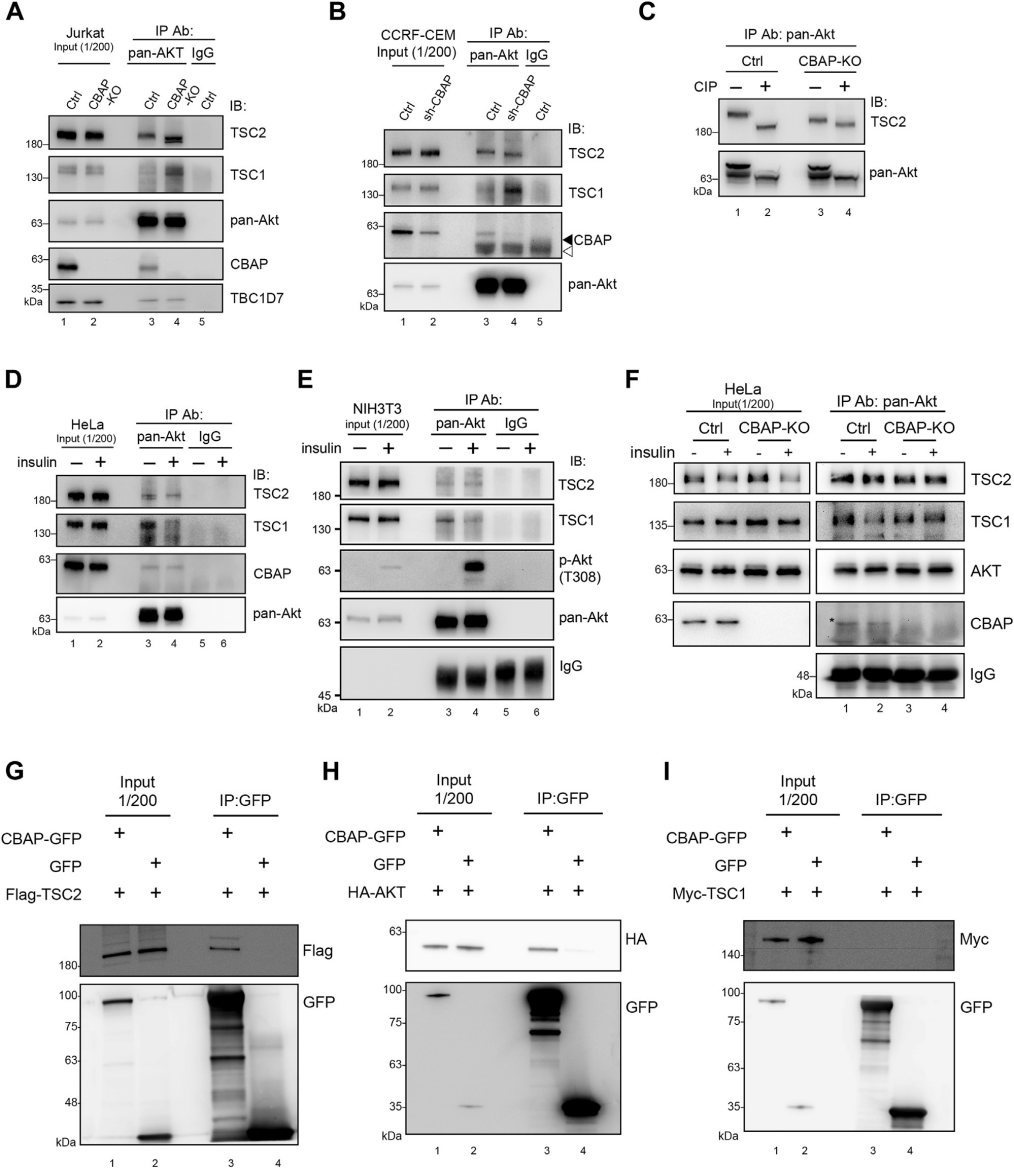
**CBAP can form complexes with Akt and TSC2 and affect the level of TSC1 within the Akt immune complexes.** Akt-associated TSC1 was increased while CBAP was depleted. Lysates from Jurkat (*A*) or CCRF-CEM (*B*) T-ALL leukemia cell lines were immunoprecipitated with anti-pan-Akt antibodies, followed by immunoblotting with indicated antibodies. “◄” indicates the specific signal of the antibody and “◁” indicates a non-specific signal. *C*, phosphorylation-dependent mobility shift of TSC2 and Akt proteins. Pan-Akt immunoprecipitates from Jurkat cell lysates were treated with or without calf intestinal alkaline phosphatase (CIP) and then subjected to immunoblotting for TSC2 and pan-Akt, respectively. Akt was included as an internal control. *D* and *E*, HeLa (*D*) or NIH3T3 (*E*) cells were serum-starved for 24 h (−), before being stimulated with insulin (1 μM, 15 min) (+). After cell lysis, the lysates were subjected to immunoprecipitation with pan-Akt antibody and subjected to immunoblotting. *F*, parental and CBAP-KO HeLa cells were serum starved for 24 h (−) or further stimulated with insulin (1 μM, 15 min) (+). Equal amount of cell lysates was subjected to IP and Western blotting as described in panels *D*. *G–I*, HEK293T cells were transiently co-transfected with GFP or CBAP-GFP and Flag-TSC2 (*G*), HA-Akt1 (*H*) or myc-TSC1 (*I*). Cell lysates were immunoprecipitated with anti-GFP antibody and probed with tag-specific antibodies in a Western blot analysis.

In our previous study, we demonstrated the crucial role of CBAP in transducing Akt-mediated Rheb-mTORC1 signaling in Jurkat and HeLa cells (32). To further understand how CBAP participates in Akt/Rheb/mTORC1 signaling, we explored the possibility of interaction among these signaling molecules. As CBAP knockout (KO) Jurkat cells exhibited compromised activities of Akt signaling, we first compared the protein complexes that could be brought down by antibodies against each signaling component, such as Akt, TSC1, TSC2, TBC1D7, and CBAP, from both control and CBAP KO Jurkat cells. Interestingly, we observed a significant difference in the complex composition only with immune complexes brought down by anti-pan Akt antibody (Fig. 1*A*), but not with antibodies against TSC1, TSC2, TBC1D7, and CBAP (Fig. S1, *A–D*). In the Western blot analysis, we observed the presence of CBAP and TSC2 but only a few TSC1 in the Akt immunoprecipitates of control Jurkat cells (Fig. 1*A*, lane 3). However, in CBAP knockout cells, the Akt complexes contained a significantly greater amount of TSC1 (Fig. 1*A*, lane 4). It is worth noting that although these signaling molecules were abundant in Jurkat cells, only a minor fraction of TSC1, TSC2, TBC1D7, and CBAP proteins could be precipitated by the anti-pan Akt antibody (Fig. 1*A*). We also examined the human T-ALL cell line CCRF-CEM and found that the anti-pan Akt immune complexes in these cells contained a similar level of TSC2, but significantly more TSC1 in CBAP knockdown CCRF-CEM cells (32) (Fig. 1*B*, lane 4). Interestingly, we observed a mobility shift of the TSC2 band on the gel in the Akt immune complexes, which was not seen in the total cell lysates (Fig. 1*A*, lanes 1&2) or immune complexes precipitated with anti-TSC1, anti-TSC2 or anti-TBC1D7 antibodies (Fig. S1, *A–C*) from control or KO cells. Moreover, the migration of TSC2 in the Akt complexes isolated from both control and knockout cells could be further downshifted by phosphatase treatment (Fig. 1*C*). These results suggest that the lack of CBAP led to a decrease in the phosphorylation status or levels of Akt-bound TSC2, which is different from the majority of TSC2 proteins that are unbound with Akt (Fig. 1*A*, lanes 1&2). To investigate the potential physiological role of these Akt/TSC1/2 complexes, we examined the stability of TSC1-TSC2 interaction in Akt immune complexes upon insulin stimulation. As depicted in Figure 1*D* of HeLa cells and 1E of NIH3T3 cells, the pan-Akt antibody pulled down comparable levels of CBAP and TSC2, but significantly fewer TSC1 in the presence of insulin (lane 3) and more TSC1 in the absence of insulin (lane 4). The mobility shift of TSC2 bands on the gel was not as noticeable as in T-ALL cells. To further confirm that CBAP is also necessary for decreasing TSC1 binding in responding to insulin stimulation, we directly compared Akt-associated TSC1 in both control and CBAP-KO HeLa cells with or without insulin addition. As shown in Figure 1*F*, insulin stimulation always decreased TSC1 in Akt immunocomplexes in control cells (lanes 1 and 2; 1.0: 0.45 ratio in Fig. S1*F*), however, insulin failed to decrease TSC1 protein levels in CBAP-KO HeLa cells (lanes 3 and 4; 0.67: 0.72 ratio in Fig. S1*F*). Therefore, in HeLa cells, CBAP proteins are required, but not sufficient for insulin stimulation-induced TSC1 dissociation from Akt immune complexes. This implies that successful dissociation of TSC1 from Akt/TSC2 complexes requires both CBAP and insulin signals.

Next, to investigate whether CBAP forms complexes with other signaling molecules by direct interaction, we conducted a series of pulldown experiments using exogenously expressed tagged CBAP. HEK293T cells were transfected with either GFP-tagged CBAP or control GFP proteins along with Flag-TSC2 (Fig. 1*G*), HA-Akt (Fig. 1*H*), or Myc-TSC1 (Fig. 1*I*), and then subjected to pulldown with anti-GFP antibody followed by Western blot analysis. Our results revealed that GFP-tagged CBAP (lane 3), but not GFP protein (lane 4), formed complexes with TSC2 (Fig. 1*G*) and Akt (Fig. 1*H*), but not TSC1 (Fig. 1*I*). Therefore, CBAP not only directly contacts Akt and TSC2 but also contributes to the stability of TSC1/TSC2 in Akt immune complexes.

### The presence of endogenous CBAP on lysosomes

**Figure 2:**
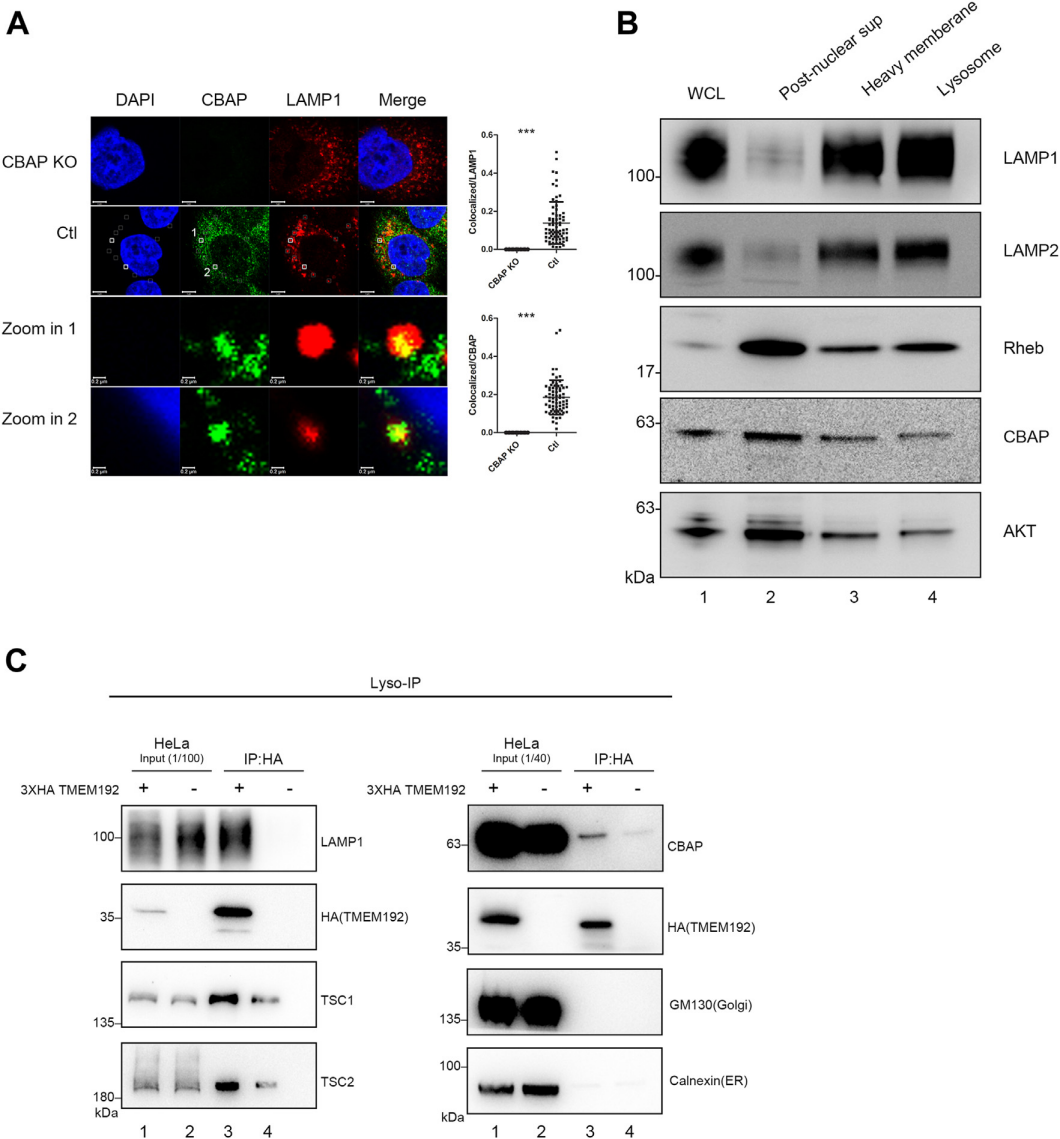
**CBAP exists on the lysosome.***A*, the lysosomal localization of CBAP in Ctrl or CBAP KO HeLa cells was investigated by co-staining with anti-CBAP and lysosomal-specific (Lamp1) antibodies. DAPI (*blue*) showed nucleus staining (*upper left*); CBAP staining was shown as *green* (*upper middle*); Lamp1 staining shown as *red* (*upper right*); The presence of a white hollow square indicated a correlated event between CBAP and LAMP1. Upon zooming in the white hollow square, colocalization between CBAP and LAMP1 was observed. Scale bars, 5 μm. Pearson correlation coefficient was measured for colocalized signal over CBAP or over Lamp1 from 5 to 10 representative confocal images in three independent experiments. These quantified data were shown in the right part. *B*, CBAP is present in lysosome-enriched fractions. Cell lysates were fractionated through the lysosome enrichment protocol as described in Materials and Methods. Aliquots representing equal protein were loaded to each lane and subjected to PAGE analysis and Western blotting. WCL, whole cell lysates. *C*, the presence of CBAP in the highly purified lysosome preparation by lyso-IP. Using anti-HA antibody, lysosomes from cells expressing 3xHA-TMEM192 (+) or not (−) were isolated and eluted by HA peptides as described in Materials and Methods. An equal amount of proteins was subjected to PAGE analysis and Western blotting. Individual target protein was detected by specific Ab as indicated.

The TSC2-dependent suppression of the Rheb-mTORC1 signaling pathway primarily occurs on the lysosomal membrane (3, 33). As CBAP is required for Akt-mediated activation of mTORC1 signaling and forms a complex with Akt and TSC2, we investigated whether CBAP is also present on the lysosomal membrane using various techniques. Firstly, we performed co-localization analysis by confocal microscopy using antibodies against human CBAP (Fig. S2) and the lysosomal marker LAMP1, and observed that staining of CBAP showed very well-demarcated puncta images, which were absent in CBAP-KO cells, and around 20% of CBAP co-localized with 13% of LAMP1 (Fig. 2*A*, right part). To further confirm this observation, we performed two additional biochemical analyses of lysosome fractions. Firstly, cell extracts were subjected to differential centrifugation to obtain nuclei-free, heavy membrane, and lysosome-enriched fractions by Calcium precipitation using a Sigma-Aldrich lysosome isolation kit (see Materials and Methods) for Western blot analysis. CBAP was detected in the nucleus-free cell lysates, heavy membrane, and lysosome-enriched fractions, which showed a similar distribution pattern to Akt and Rheb (Fig. 2*B*). LAMP1 and LAMP2, as lysosomal marker proteins, were highly enriched in lysosomes (Fig. 2*B*). Although both CBAP and Akt were not enriched specifically in lysosomes likes TSC1, but they are present in various membranous organelles equally well. Secondly, we established the 3xHA-TMEM192 overexpressing HeLa cells and purified lysosomes using Lyso-IP with anti-HA tag antibodies, and HA-immunoprecipitated lysosomes were then eluted by HA peptides (34). As shown in Figure 2*C*, CBAP was clearly detected in the highly purified lysosomes, along with other lysosomal marker proteins, such as LAMP1, TMEM192, or other components of the Akt signaling pathway, such as TSC1 and TSC2 (Fig. 2*C*). GM130 (a Golgi marker) and Calnexin (an ER marker) were included as negative controls for isolated lysosomes (Fig. 2*C*, bottom two rows). Therefore, based on our confocal images and subcellular fractionation experiments, CBAP appears to have a wide distribution in heavy membranes, including the lysosomal membrane.

### Lysosome-targeted CBAP stimulates mTORC1 signaling dependent on TSC2 interaction

To identify the specific region of CBAP that interacts with Akt and TSC2, we expressed GFP-tagged full-length (FL) and truncated mutants of CBAP proteins with Flag-TSC2 and myc-Akt in CBAP-knockout HEK293T cells (Fig. S3*A*). In an initial mapping experiment, we found that FL, M1, and M6 mutants were able to interact with TSC2, but not M4 and M7. On the other hand, FL, M1, and M7 mutants were able to interact with Akt, but not M4 and M6 (Fig. S3*B*). These results suggest that TSC2 and Akt bind to distinct regions of the CBAP protein. Importantly, FL and M1 were able to rescue TSC2 phosphorylation at T1462 in CBAP-KO HEK293T cells, whereas M6 and M7 were not (Fig. S3*B*). This strongly suggests that simultaneous interaction with TSC2 and Akt may be essential for CBAP to facilitate Akt-dependent TSC2 phosphorylation.

**Figure 3:**
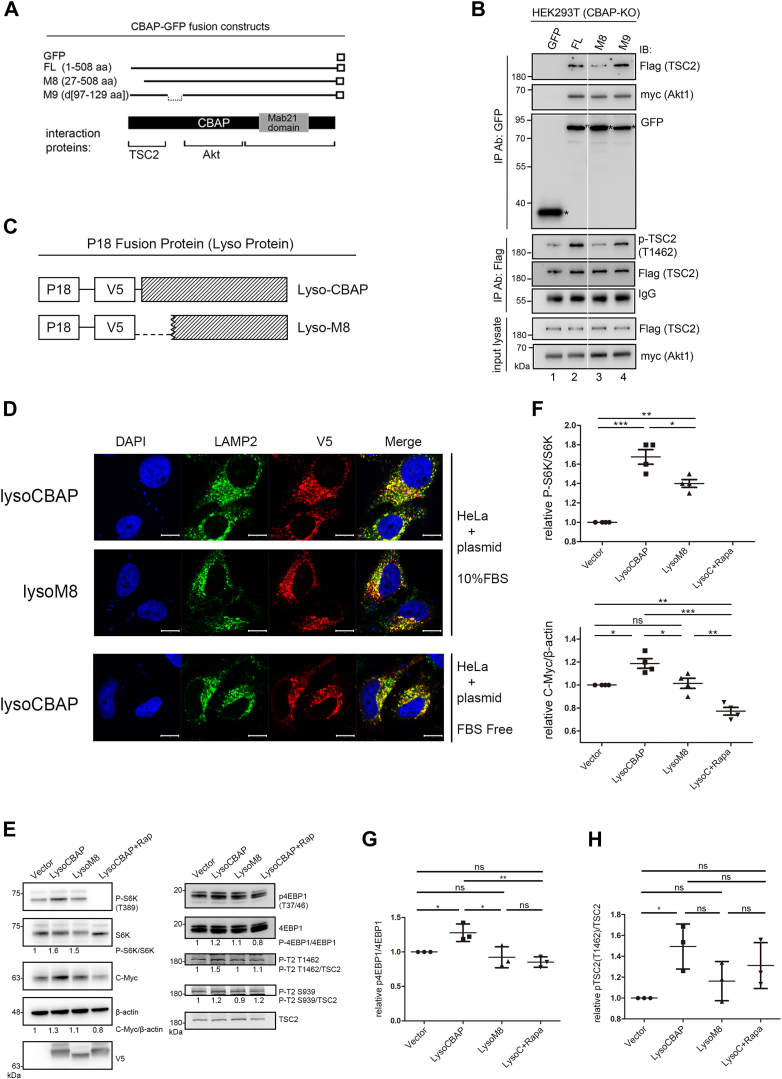
**Lyso-CBAP drives mTORC1 signaling activation *via* CBAP-TSC2 interaction.***A*, schematic representation of various truncated CBAP-GFP constructs and the Akt and TSC2-interacting domains of CBAP. *B*, the N-terminus 26 amino of CBAP is required for CBAP and TSC2 interaction. The truncated CBAP-GFP constructs were co-transfected with myc-Akt1 and Flag-TSC2 into CBAP-KO HEK293T cells. Anti-GFP or anti-Flag immunoprecipitates were blotted with the indicated antibodies. Images of lanes 1 and 2 were grouped together with lanes 3 and 4 from the same gel and same exposure (splicing sites were shown as a *white line*) for the sake of brevity. *C*, schematic representation of Lyso-tagged proteins Lyso-CBAP and Lyso-M8. P18 represents the amino terminus of p18/LAMTOR1, which served as a lysosomal anchor. V5 tags were used for the detection of the transfected genes. *D*, expression of p18-tagged CBAP and M8 was highly localized on lysosomes. HeLa cells were transiently transfected with either Lyso-CBAP, or Lyso-M8 in complete medium prior to immunofluorescence staining with the indicated anti-V5 and anti-Lamp2 antibodies (*top two rows*). Scale bars, 10 μm. The images of V5-tagged LysoCBAP under FBS starvation were also presented (*bottom row*). Scale bars, 10 μm. *E*, Lyso-CBAP can activate mTORC1 signaling in the absence of serum. HeLa cells expressing Vector, LysoCBAP or LysoM8, with or without rapamycin treatment (1 nM), were subjected to signaling analysis by probing with specific antibodies as indicated. Beta-actin serves as a loading control, and V5 tag Ab detects the expression of transfected genes. One representative figure is shown from three or four independent repeats. *F–H*, the relative intensities of p-S6K(T389)/S6K and c-Myc/β-actin (panel *F*), p-4EBP1(T37/46)/4EBP1 (panel *G*), and p-TSC2(T1462)/TSC2 (panel *H*) of each sample were measured and then normalized further with the value in vector sample. Three or four repeats have been done. One-way ANOVA was used to calculate statistical significance; ns, nonsignificant; ∗*p* < 0.05; ∗∗*p* < 0.01; ∗∗∗*p* < 0.001.

We then generated two small deletion mutants, M8 and M9, to further map the interaction region with TSC2 (Fig. 3*A*). Again, we found that expression of FL and M9 mutants of CBAP formed complexes with TSC2 and rescued phosphorylation (Fig. 3*B*, lanes 2 and 4), while the M8 mutant slightly reduced interaction with TSC2 and significantly hindered T1462 phosphorylation of TSC2 (Fig. 3*B*, lane 3). Since both M8 and M9 mutants were capable of interacting with Akt, this result indicates that the N-terminal 26 amino acids of CBAP are required for the interaction of CBAP with TSC2 and successful TSC2 phosphorylation by Akt (Fig. 3*B*).

To determine whether the interaction of CBAP with TSC2 on the lysosomal membrane is crucial for its function in mTORC1 activation, we created lysosome-anchored wild-type and M8 CBAP proteins and compared their ability to activate mTORC1 signaling in the absence of serum. As only a small proportion of wild-type CBAP (∼13%) is colocalized with Lamp1, and presumably locates on lysosomes, we fused the protein to the amino terminus of p18/LAMTOR1 (3) (Lyso-CBAP and Lyso-M8) to maximize its localization to lysosomes (Fig. 3*C*). Immunofluorescence confocal microscopy analysis confirmed the predominant lysosomal localization of these Lyso-fusion proteins in HeLa cells, with Lyso-CBAP being up to ∼90% localized onto lysosomes and Lyso-M8 to ∼80% (Fig. 3*D*, top two rows and Fig. S3*C*). Lysosome-localized Lyso-TSC2 was used as a documented comparison (3) and was found to be ∼85% on lysosomes in a normal serum medium (Fig. S3*C*). In the absence of serum, expression of lysosome-localized Lyso-CBAP increased phosphorylation of TSC2(T1462), S6K1(T389), 4EBP1(T37/46), and c-Myc expression, while Lyso-M8 had less or no effect (Fig. 3, *E*–*H*). These signal activations were sensitive to Rapamycin, as rapamycin treatment (1 nM) almost abolished the increments (Fig. 3, *E*–*H*, lanes lysoCBAP+Rapa), indicating that they were activated through the mTORC1 pathway. Therefore, these results strongly suggest that the localization of CBAP on the lysosomal membrane actively participates in TSC2-dependent mTORC1 signaling transduction.

### CBAP suppresses the GAP function of TSCC in the Akt complex

**Figure 4:**
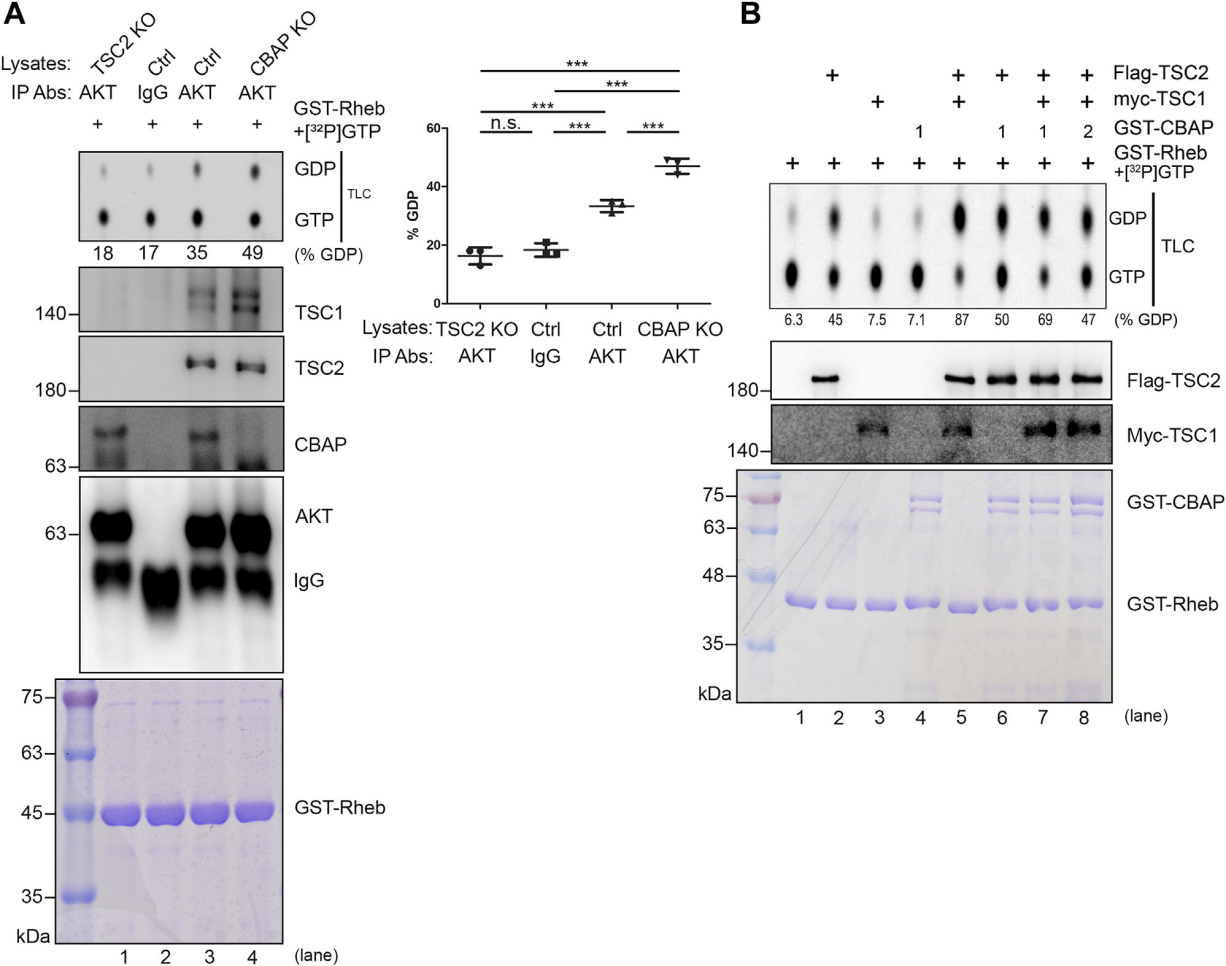
**CBAP decreases the GAP activity in anti-Akt targeted TSC1/2 population.***A*, increased TSC2 GAP activity of Akt immunocomplexes in CBAP-KO Jurkat cells using thin layer chromatography (TLC) method. Total Jurkat cell lysates (TSC2-KO, Ctrl and CBAP-KO) were immunoprecipitated with anti-pan-Akt antibodies, or a control IgG antibody, before being subjected to a TSC2 GAP assay using recombinant GST-Rheb preloaded with [α-^32^P]-radiolabeled GTP. The radioactivity of each spot was measured and the percentage GTP-to-GDP conversion was calculated. Protein levels of TSC1, TSC2, CBAP and Akt in each immunoprecipitate were monitored by Western blotting. Coomassie blue staining showed equal loading of the GST-Rheb recombinant proteins in each reaction. One representative data from three independent experiments was shown. Quantified GTP-GDP conversion rates were shown in the right part of the panel. One-way ANOVA was used to calculate statistical significance; ns, nonsignificant; n.s., none significant; ∗∗∗*p* < 0.001. *B*, dose-dependent suppression of TSC2 GAP activity by CBAP using *in vitro* translated recombinant TSC1/TSC2 complexes. IVT-synthesized Flag-TSC2 and myc-TSC1 were immunoprecipitated with tag-specific antibodies, and then subjected to an *in vitro* TSC2 GAP assay in the absence or presence of different amounts of recombinant GST-CBAP. Rheb-bound GTP and GDP were resolved by TLC. The percentage GTP-to-GDP conversion is indicated below the lanes. Each lane of reaction was also subjected to SDS-page analysis and probed with the indicated antibodies. Coomassie blue staining showed equal loading of GST-Rheb and various amounts of GST-CBAP proteins.

To stabilize and enhance TSC2 GAP activity *in vitro* and *in vivo*, the association of TSC1 with TSC2 is crucial (6, 7, 35). Therefore, the increased level of TSC1 associated with the Akt immune complexes in CBAP-deficient Jurkat cells could have a significant impact on the GAP activity of TSC2. To investigate whether the absence of CBAP altered the GAP activity of the Akt immune complexes, we conducted GAP activity assays using ^32^P-GTP-loaded GST-Rheb with anti-Akt immunoprecipitates from control and CBAP-deficient Jurkat cells and revealed a conversion of 35% and 49% of GTP-Rheb into GDP-Rheb, respectively (Fig. 4*A*, lanes 3 and 4). We also carefully monitored the loading of GST-Rheb (bottom panel, Coomassie blue staining), and the protein levels of Akt, CBAP, TSC2, and TSC1 in each immune-precipitated sample (panels with specific antibodies in Fig. 4*A*). To rule out the possibility that the anti-pan Akt antibody likely brings down some unknown factors that can convert Rheb-GTP into Rheb-GDP, we included a TSC2-KO Jurkat cell line (see cell lysates input controls in Fig. S4) in the panel. As shown in Figure 4*A*, lane 1, anti-pan Akt antibody brought down similar levels of Akt and CBAP, but no TSC2 (due to gene knockout) and no TSC1 (due to no direct interaction with Akt or CBAP), and these immune precipitates showed only background level of GAP activity (similar to control antibody in lane 2) toward Rheb-GTP provided, suggesting it is TSC2 contributes to the GAP activity in our Akt immunoprecipitates (Fig. 4*A*, lanes 3 and 4). We therefore propose that the presence of CBAP could suppress the GAP activity of the Akt-bound TSC1-TSC2 complexes, which are precipitated by anti-pan Akt antibody. To further explore this hypothesis, we conducted an add-on experiment to explore whether CBAP can inhibit the GAP activity in the *in vitro* TSC2 GAP reaction (3). The assay reaction included recombinant Flag-TSC2 with or without recombinant myc-TSC1 in combination with bacterially-expressed CBAP. Our data showed that TSC2 alone resulted in approximately 45% conversion of GTP-Rheb to GDP-Rheb (Fig. 4*B*, lane 2), while the conversion increased to around 87% when both TSC1 and TSC2 were present (Fig. 4*B*, lane 5), consistent with a previous report (6). The inclusion of CBAP, however, reduced the conversion to approximately 69% and 47% with 1 μg and 2 μg of CBAP, respectively (Fig. 4*B*, lanes 7 and 8). Careful controls of protein loading in each GAP reaction were also provided underneath the reaction panel. Taken together, these data strongly suggest that the association of CBAP with TSCC suppresses the GAP activity of TSCC toward the Rheb GTPase.

### CBAP competes with TSC1 for TSC2 HBD domain binding through its hydrophobic N-terminus domain

**Figure 5:**
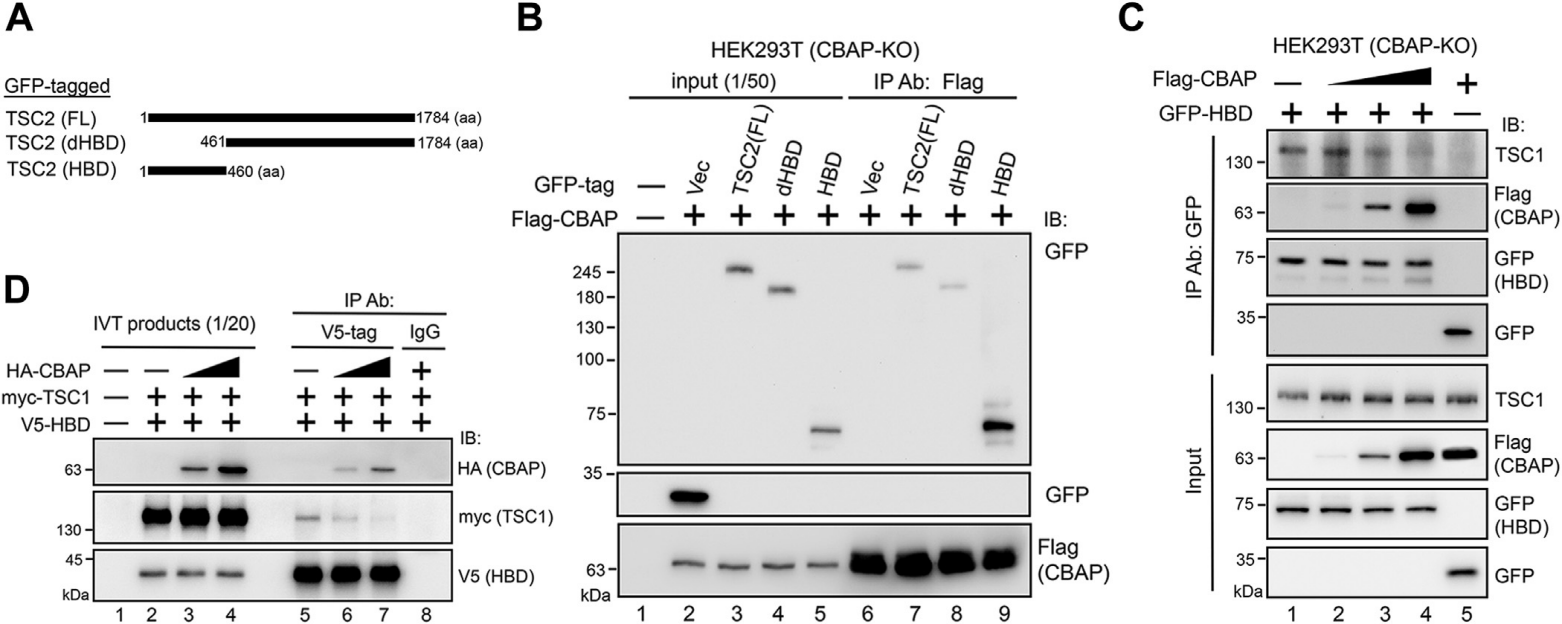
**CBAP competes with TSC1 for TSC2 HBD domain binding through its N-terminus domain.***A* and *B*, CBAP associates with TSC2 protein *via* its HBD domain. Cells were co-transfected with Flag-CBAP and GFP-tagged vector or the indicated truncated TSC2 plasmids (*A*). After lysis, cell lysates were used for immunoprecipitation of Flag-CBAP and then subjected to immunoblotting using anti-GFP antibody (*B*). HBD represents the hamartin-binding domain of TSC2. The dHBD construct reflects deletion of the HBD from TSC2. *C* and *D*, competition for HBD binding between CBAP and TSC1 *in vivo* (*C*) and *in vitro* (*D*). *C*, CBAP-KO HEK293T cells were transiently co-transfected with GFP-HBD and different amounts of Flag-CBAP plasmids. Cell lysates were immunoprecipitated with an anti-GFP antibody, and then endogenous levels of TSC1 were monitored. *D*, HA-CBAP, myc-TSC1, and V5-HBD proteins were co-synthesized using an IVT system and then subjected to GFP-based immunoprecipitation and immunoblotting analyses.

Why does CBAP reduce the GAP activity of TSC1/2 complexes? Could it be possible that the binding of CBAP to TSC2 affects the interaction of TSC2 with TSC1, thereby inhibiting the functionality of TSCC? To address this question, we investigated whether CBAP and TSC1 bind to the same domain on TSC2. Previous studies have shown that TSC1 binds to the hamartin-binding domain (dHBD) of TSC2 (36). We expressed various TSC2 mutant proteins, such as full-length TSC2 fused with GFP (FL), TSC2 lacking the HBD, and the TSC2-HBD alone (HBD), and tested their associations with full-length CBAP (Fig. 5*A*). Deletion of the HBD from TSC2 slightly reduced its ability to bind CBAP (Fig. 5*B*, lane 8) compared to the full-length control protein (lane 7). However, the HBD alone (lane 9) bound Flag-CBAP much more strongly than either the FL or dHBD constructs. These findings suggest that while the HBD in the N-terminus of TSC2 contributes to the interaction with CBAP, other regions of TSC2 may also be involved.

To further investigate the effect of CBAP on the interaction between TSC2 and TSC1, we expressed equal amounts of exogenous GFP-tagged HBD with increasing amounts of Flag-tagged CBAP in CBAP-deficient HEK293T cells. They then subjected the whole cell lysates to anti-GFP precipitation. In the absence of CBAP proteins, HBD-associated immunoprecipitates brought down endogenous TSC1 proteins (Fig. 5*C*, lane 1). However, in the presence of increasing amounts of CBAP, the HBD-associated immunoprecipitates brought down progressively less TSC1 protein (Fig. 5*C*, lanes 2–4). These results suggest that the presence of CBAP reduces the association of TSC1 with TSC2, possibly by competing with TSC1 for binding to TSC2 and thus inhibits the functionality of TSCC. We conducted an *in vitro* competition experiment with in vitro-synthesized TSC1 and HBD, as well as bacterially-produced CBAP to further investigate this hypothesis. When CBAP was absent, TSC1 was precipitated by HBD (Fig. 5*D*, lane 5). However, as we added increasing amounts of recombinant CBAP protein, less TSC1 was brought down (lanes 6 and 7). These experiments strongly suggest that CBAP can directly compete with TSC1 for binding to the HBD of TSC2.

### Perturbing the interaction between CBAP and TSC2 reduces Rheb-GTP and impedes cell growth

**Figure 6:**
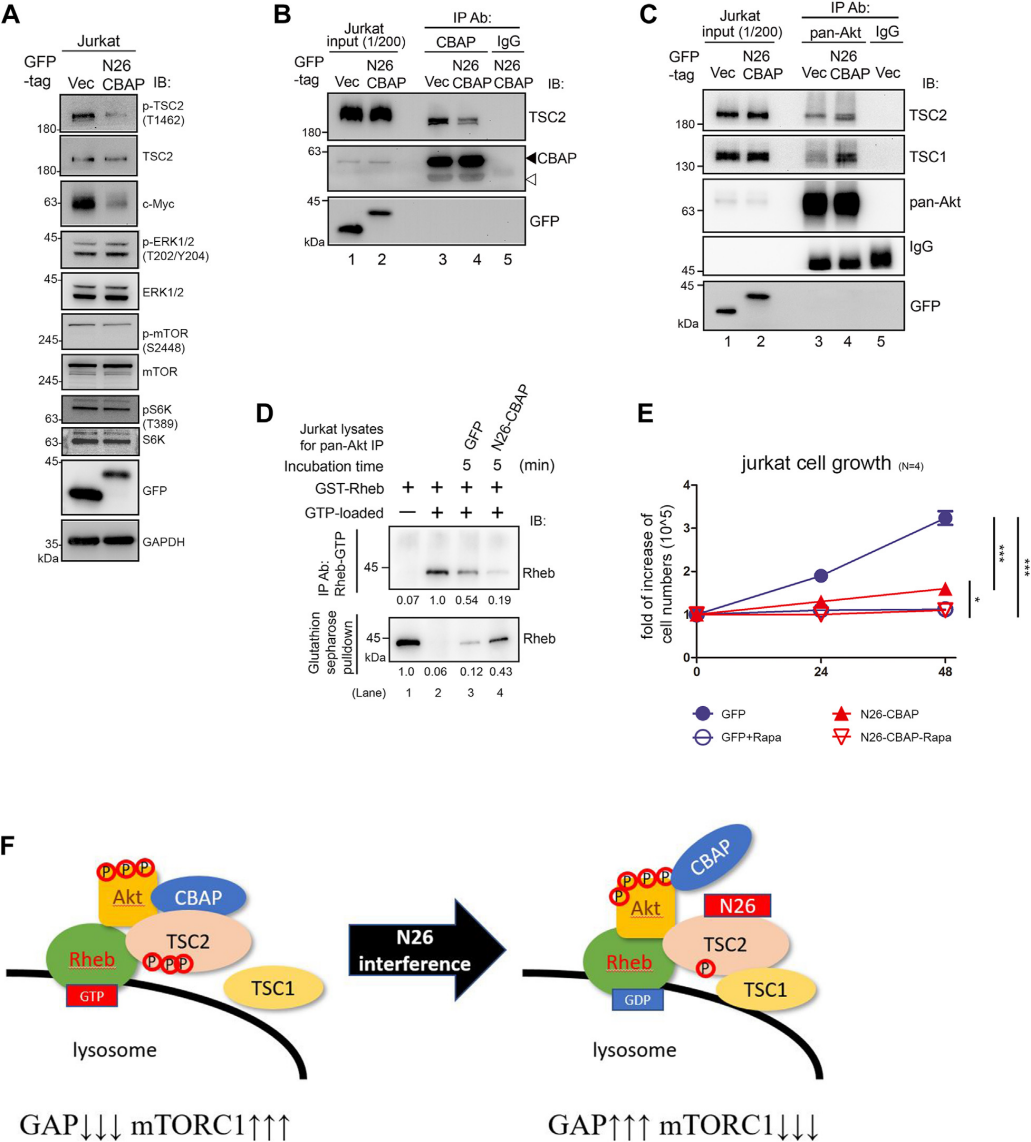
**Interference of CBAP and TSC2 interaction impairs tumor cell growth.***A–C*, N26 efficiently inhibits mTORC1 signaling in Jurkat cells by interfering with the CBAP and TSC2 interaction. Lysates of GFP-N26-CBAP-expressing cells were subjected to immunoblotting (*A*) or were immunoprecipitated with anti-CBAP antibody (*B*). *C*, expression of N26-CBAP increases the TSC1 within Akt immune complexes. GFP (+) vector (Vec) cells and GFP-N26-CBAP-expressing cells were both sorted by flowcytometry and subjected to immunoprecipitation with anti-pan-Akt antibody. The immunoprecipitates were subjected to analysis described in Figure 1*A*. *D* and *E*, GFP-N26-CBAP increased the TSC2 GAP activity and inhibited Jurkat cell growth. Cell lysates of GFP(+) cells were used for *In vitro* GAP assays using non-isotope GTP-loaded GST-Rheb as substrates and anti-GTP-Rheb specific antibody to distinguish GTP-Rheb *versus* GDP-Rheb (*D*). Viable cell numbers in the GFP (+) population with or without rapamycin treatment were determined by trypan blue exclusion staining at indicated time points (*E*). One-way ANOVA was used to calculate statistical significance; ∗*p* < 0.05; ∗∗∗*p* < 0.001., n = 4. *F*, model of N26-CBAP interference of mTORC1 signaling in Jurkat cells *via* regulating stability of TSC1/2-Akt complexes. N26-CBAP perturbs CBAP-TSC2 interaction, leading to an increase of TSC1 binding to TSC2-Akt complex, increase of Rheb-GTP hydrolysis, and decrease of mTORC1 signaling.

We conducted experiments to investigate the potential dominant negative effect of overexpression of the N26 peptide on the function of endogenous CBAP proteins. M8-CBAP, which lacks N26 amino acid sequences, significantly decreased interaction with TSC2. Intriguingly, we observed that N26 peptides could significantly diminish phosphorylation of TSC2, mTOR (by approximately 30%, as seen in Figs. S5*A*), and S6K1 (by approximately 20%, as seen in Fig. S5*B*), but not ERK1/2 phosphorylation, leading to reduced levels of c-Myc protein in Jurkat cells (as shown in Fig. 6*A*). In addition, the amount of TSC2 co-immunoprecipitated with endogenous CBAP was decreased in N26-expressing cells, indicating interference with the binding between full-length CBAP and TSC2 (as seen in Fig. 6*B*). Furthermore, when we conducted a co-immunoprecipitation experiment in N26-expressing cells with anti-pan Akt antibody, we detected faster-migrating TSC2 and increased levels of TSC1 in Akt-immune complexes (as seen in Fig. 6*C*), as well as enhanced TSC2 GAP activity (as shown in Fig. 6*D*). Moreover, the expression of N26-GFP led to a significant decrease in Jurkat cell proliferation compared to control GFP cells (*p*-value < 0.001, n = 3). Interestingly, the proliferation of both types of cells could be further inhibited by treatment with mTORC1 inhibitor Rapamycin (*p*-value < 0.001 for GFP cells and *p*-value < 0.01 for GFP-N26 cells) (Fig. 6*E*). Thus, by overexpressing N26 peptides, we created a cellular condition that resembles CBAP deficiency in Jurkat cells. These results strongly indicate that the N26-CBAP peptide can indeed exert its dominant negative effect on CBAP/TSC2 complexes, leading to a significant reduction in tumor cell proliferation.

These findings further reinforce the correlation between the stability of Akt/CBAP/TSC2 immune complexes and the activity of Rheb/mTORC1 signaling. The biological significance of the presence of and regulation of the Akt/CBAP/TSC2 complexes in response to various growth factors and external stimuli warrants extensive investigation.

## Discussion

The primary objective of this study is to delve into the intricate mechanisms through which CBAP intricately participates in the regulation of Rheb-mTORC1 signaling *via* Akt. In a prior investigation, we robustly demonstrated that the absence of CBAP exerts a powerful inhibitory effect on Akt-mediated TSC2 phosphorylation and the consequential activation of Rheb/mTORC1. This outcome translates into a notable reduction in cell growth and leukemogenicity in Jurkat cells (32). At a mechanistic level, our study showcased the essential role of CBAP in both *in vivo* and *in vitro* TSC2 phosphorylation catalyzed by activated Akt proteins (32). Furthermore, we meticulously observed a reduction in the translocation of TSC2 away from the lysosomal membrane in CBAP-KO HeLa cells, closely aligning with the hypothetical model proposed by Menon *et al.* (32). Intriguingly, we also made the noteworthy observation that the co-localization of TSC2 proteins with lysosomal markers, such as Lamp1 or lysosome-specific dyes, did not completely vanish. In simpler terms, even with insulin stimulation, capable of fully activating Rheb/mTORC1 signaling, akin to cells lacking TSC2 protein expression, the lysosomal co-localization of TSC2 persisted at approximately 20 to 30% (as depicted in Fig. 6). This insight strongly suggests that the lysosomal translocation mechanism may represent just one facet of the multifaceted processes by which PI3K-Akt signaling propels Rheb-GTP loading, while concurrently mitigating the counteractive effects of TSC2 on Rheb. In this report, we delve into the potential significance of protein-protein interactions in Akt-dependent TSC2 phosphorylation and the activation of Rheb/mTORC1. Our comprehensive dataset, gathered through a blend of immunological and biochemical analyses, provides robust evidence to support the notion that CBAP exerts a significant influence on the stability of the TSC1-TSC2 interaction within the Akt complexes. Furthermore, CBAP plays a pivotal functional role in suppressing the GAP activity of TSCC, ultimately leading to an elevation in GTP-bound Rheb levels, thereby amplifying mTORC1 signaling.

Our research provides compelling evidence that CBAP exerts a diminishing effect on the presence of TSC1 within Akt complexes in Jurkat cells. Furthermore, our experimental findings consistently illustrate that full-length CBAP competes effectively with TSC1 for TSC2 binding, both *in vivo* and *in vitro*. Based on these insights, we posit that CBAP's competitive interaction extends beyond the promotion of TSC1/TSC2 removal from lysosomes to its active participation in displacing TSC1 within Akt/TSC2 complexes on lysosomal membranes. This competitive interplay results in a reduction of TSC2's GAP activity towards Rheb, thereby facilitating the accumulation of GTP-bound Rheb on the cellular membrane and, consequently, the activation of mTORC1 signal transduction. Our hypothesis is further substantiated by additional experimental observations. First, we have observed the presence of endogenous CBAP on the lysosomal membrane, a pivotal site for initiating Rheb/mTORC1 signaling. Second, we find that lysosomal-anchored CBAP can effectively stimulate mTORC1 signaling, particularly in serum-starved conditions, potentially by enhancing the dissociation of TSC1 within membrane-bound Akt/TSC2 complexes. Finally, we have demonstrated that the overexpression of N26-CBAP peptides can dominantly interfere with CBAP-TSC2 interactions in wild-type Jurkat cells, effectively replicating the growth retardation observed in CBAP KO Jurkat cells (32). Collectively, our data strongly underscore the pivotal role of protein-protein interactions in governing Akt-mediated TSCC functional regulation.

In this study, we conducted an in-depth exploration of the interactions among signaling components within the Akt/CBAP/TSC2/Rheb pathway, a system abundantly present in mammalian cells. Notably, our immunoprecipitation experiments utilizing Akt and CBAP antibodies unveiled an intriguing revelation: a mere fraction, estimated to be less than 1% of the total, of TSC2-TSC1 complexes engages with either Akt or CBAP. This implies that the majority of TSC1-TSC2 complexes do not establish interactions with Akt or CBAP. Furthermore, our investigations have spotlighted a critical detail: only CBAP proteins capable of simultaneous association with both TSC2 and Akt can effectively rescue the Akt-mediated TSC2 maximal phosphorylation at T1462 in CBAP-KO Jurkat cells. Building on this observation, we formulated a hypothesis: the pool of Akt-free TSC1-TSC2 complexes, represented by the supernatant fraction post-Akt immunoprecipitation, predominantly houses under-phosphorylated TSC2 proteins. It is within the Akt/CBAP complexes that CBAP exerts its influence, facilitating maximal TSC2 T1462 phosphorylation and the disruption of TSC1-TSC2 integrity. This phenomenon appears to be exclusive to Akt/CBAP/TSCC complexes and is not replicated in the overall pool of TSCC complexes. Consequently, this hypothesis provides an explanation for why TSC2-TSC1 complexes precipitated with TSC1 or TSC2 antibodies (as depicted in Fig. 4*D* and (3)) exhibit no discernible changes in GAP activity in response to insulin stimulation, as these antibodies predominantly capture the total pool of TSC2 complexes. In summary, our findings shed light on a multifaceted regulatory mechanism governed by intricate protein-protein interactions within the Akt/CBAP/TSC2/Rheb pathway.

In our prior study, we established that the absence of CBAP expression leads to growth suppression, a reduction in Rheb/mTORC1 activity, and a noteworthy decrease in the translocation of TSC2 proteins to lysosomes. These findings align with earlier reports by Menon (3) and Cai (19). However, in this current report, we introduce additional data that hints at an alternative mechanism through which CBAP may activate the Rheb/mTORC1 pathway. Specifically, CBAP appears to inhibit the GAP activity of TSC2 by dissociating TSC1—a novel mechanism that warrants further comprehensive exploration. It remains to be ascertained to what extent this dissociation of TSC1 contributes to the activation of Rheb/mTORC1 and whether it operates independently or in conjunction with the lysosomal translocation mechanism that we previously documented.

## Experimental procedures

### Cell line

Jurkat (TIB-152), CCRF-CEM (CCL-119), and HeLa (CCL-2) cell lines were obtained from the American Type Culture Collection (ATCC). These parental and their derived cell lines were cultured according to ATCC instructions. TSC2-KO Jurkat cell line was generated in this research by CRISPR-Cas9 technology. CBAP-KO Jurkat cell lines were described previously (32).

### Antibodies and reagents

Anti-Flag M2 affinity gel was used for immunoprecipitation of Flag-tagged proteins (A2220; Sigma-Aldrich). Antibodies against the following proteins were purchased for immunoprecipitation or immunoblotting: Akt1/2/3 (pan-Akt; ab179463), p-Akt1 (S473; ab81283), TSC2 (ab52936), and Rheb (ab92313) from Abcam; actin (3700), c-Myc (5650), Myc-tag (2276), TSC2 (4308), p-TSC2 (S939 and T1462; 3615 and 3617), p-ERK1/2 (T202/Y204; 4370), ERK1/2 (4695), p-mTOR (S2448; 2971), p-4E-BP1 (T37/46; 2855) and p-p70 S6 Kinase (T389; 9205), TSC1 (6935), TBC1D7 (14949), V5-tag (13202), Rheb (13879), Lamp1 (9091) all from Cell Signaling Technology; GFP (GTX113617), HA tag (GTX115044, GTX628489) and GAPDH (GTX100118) from GeneTex (Irvine, CA); GFP (632381) from Clontech; and Flag (F7425) from Sigma-Aldrich. Active-Rheb (Rheb-GTP)-specific antibody was purchased from NewEast Biosciences (26910). Anti-Lamp2 (SC-18822) was obtained from Santa Cruz. Mouse anti-CBAP monoclonal antibody has been described previously (28). Rabbit anti-CBAP polyclonal antibody was purchased from Proteintech. Calf intestinal alkaline phosphatase (CIP) was obtained from New England Biolabs. *In vitro* transcription/translation (IVT) of myc-TSC1, Flag-TSC2 or myc-Akt1 protein was achieved using a TnT T7/T3 Coupled Reticulocyte System (Promega; L5010). Bacterial recombinant T7-Rhen (Pro-308) and His-Akt1 (PSPKA-068) were purchased from ProSpec. Protein G Mag Sepharose was purchased from Sigma-Aldrich.

### Plasmids

GFP-tagged *TSC2*, *HBD-TSC2* and *dHBD-TSC2* plasmids were kindly provided by Dr Vera P. Krymskaya (University of Pennsylvania). Plasmids expressing Flag-tagged *TSC2* (#14129) and Myc-tagged *TSC1* (#12133) were obtained from Addgene. The Flag-*TSC2* plasmid was used as a template to generate a V5-tagged HBD construct. pcDNA3-*Akt1* plasmid was a gift from Dr Hsin-Fang Yang-Yen (IMB, Academia Sinica) and was used as a template to generate HA-tagged *Akt1* and Myc-tagged *Akt1* constructs. The full-length *Rheb* gene was cloned from cDNA of Jurkat cells and subcloned to generate Flag-tagged *Rheb* and GST-tagged *Rheb* plasmids. The generation of plasmids expressing full-length and truncated human CBAP has been reported previously (32). Plasmids expressing Lyso-CBAP and Lyso-M8-CBAP were constructed in-house by fusing the cDNAs of CBAP and M8-CBAP with the N-terminus fragment of p18/LAMTOR1 (3) and additional tag sequences. The cDNA sequence of the p18 anchorage peptide and additional V5 as follows:

p18: “GGGTGCTGCTACAGCAGCGAGAACGAGGACTCGGACCAGGACC-GAGAGGAGCGGAAGCTGCTGCTGGACCCTAGCAGCCCCCCTACCAAA-GCTCTCAATGGAGCCGAGCCCAAC”; V5 tag: “GGTAAGCCTATCCCTAAC-CCTCTCCTCGGTCTCGATTCTACG”. These oligonucleotides were synthesized by IDT (Coralville, IW).

### Transient transfection of plasmids

Suspension cells (1 × 10^7^) were transiently transfected with 30 μg of plasmids by electroporation using the Gene Pulser II system (Bio-Rad) at 200 V, 975 μF, and infinite resistance. Adherent cells were transfected with the indicated plasmids using X-tremeGENE HP transfection reagent (Roche) according to the manufacturer's instructions.

### Cell lysis, immunoprecipitation, and immunoblotting

For regular total cell lysis, cells were washed three times with cold PBS and lysed in Triton-X 100 lysis buffer containing 20 mM Tris-HCl [pH 7.5], 1% Triton X-100, 150 mM NaCl, 10 mM EDTA, 1% Na-deoxycholate, 50 mM NaF, 0.5 mM Na-orthovanadate, protease inhibitor cocktail (Sigma-Aldrich) and PhosSTOP protease inhibitor (Roche). For immunoprecipitation, cell lysates were precipitated with the indicated antibodies and protein G-Sepharose beads (GE Healthcare), and then washed with Triton-X 100 lysis buffer. Immunocomplexes were then subjected to SDS-PAGE and Western blotting. ImageJ software (Wayne Rasband, NIH) was used to quantify band intensities on the blots.

### Immunofluorescence staining, confocal microscopy and quantification of colocalization

The immunofluorescence staining experiments, and calculation of Pearson's correlation coefficient (PCC) were performed according to a protocol provided by Zeiss LSM 700 confocal microscope (Oberkochen, Germany). In brief, cells were grown on 12-mm glass coverslips and fixed with 4% paraformaldehyde in PBS for 30 min at room temperature (RT). After fixation, cells were washed three times with PBS and permeabilized in PBS containing 0.2% Triton X-100 for 30 min. After permeabilization, coverslips were washed three times and blocked overnight in a blocking buffer (PBS containing 2% bovine serum albumin (BSA)). Coverslips were then incubated overnight with primary antibody diluted in blocking buffer at 4 °C. The primary anti-CBAP antibody needed to be pre-absorbed by incubating it with cell lysates from CBAP-KO HeLa cells for 48 h at 4 °C, before changing the buffer to PBS by means of Amicon Ultra filters. Following primary antibody incubation, the coverslips were washed three times with PBS and then incubated with secondary antibodies (Thermo) for 2 h at RT. Finally, the coverslips were washed three times with PBS and slides were prepared by adding Mounting Medium (Invitrogen). For each condition, we used Zeiss LSM 700 microscope, 63× lens, and with frame size 2048 × 2048 to capture 5 to 10 representative confocal images from triplicate experiments. To calculate colocalization, we applied Pearson’s correlation coefficient to analyze a total of 30 to 200 individual cells per condition by the automatic Costes thresholding method.

### Lysosome enrichment

Lysosome enrichment was performed using the Lysosome Isolation Kit (LYSISO1, Sigma-Aldrich). Briefly, cells were harvested, homogenized with a dounce, and centrifuged at 1000*g* for 10 min. The postnuclear supernatant was then subjected to further centrifugation at 20,000*g* for 30 min. The resulting pellet was resuspended, treated with a CaCl_2_ solution, and centrifuged at 5000*g* to precipitate ER and mitochondria. Finally, the supernatant was used as enriched lysosomes and analyzed for protein composition by SDS-PAGE and Western blotting.

### Lysosome purification by Lyso-IP method

Lyso-IP was performed according to a previous report (34). Briefly, HeLa cells were transfected with 3xHA-TMEM192, a fusion protein of TMEM192 and 3xHA tag specific to lysosomes. After 24 h, the cells were harvested, homogenized with a dounce, and centrifuged at 1000*g* for 10 min to remove the nuclei. The resulting supernatant was subjected to immunoprecipitation using an anti-HA antibody. The immunoprecipitated material was eluted with HA-peptide and analyzed using SDS-PAGE and Western blotting.

### Affinity purification of GST-Rheb and *in vitro* GAP assay

GAP assay was performed based on a protocol reported previously (3, 7), but with minor modifications. In brief, bacterially expressed GST-Rheb proteins were purified with glutathione-agarose beads and then were washed three times with Rheb wash buffer (50 mM HEPES pH 7.5, 0.5 M NaCl, 0.1% Triton-X 100, 5 mM MgCl_2_, 0.005% SDS), and once in PBS. The recombinant GST-Rheb was eluted with reduced glutathione buffer (10 mM) and then subjected to buffer exchange with PBS by dialysis. The soluble GST-Rheb proteins were loaded without nucleotide or with 500 mM GTP[α-^32^P] in loading buffer (50 mM HEPES pH 7.4, 1 mM EDTA, 5 mg/ml BSA) for 1 h at 30 °C with gentle agitation, and then nucleotide binding was secured by adding 20 mM MgCl_2_ for 5 min on ice. Endogenous Akt complexes were immunoprecipitated from total cell lysates (5 mg) or the heavy membrane fraction (2 mg) using a pan-Akt antibody. After thorough washing three times with Triton-X 100 lysis buffer and once in Rheb exchange buffer (50 mM HEPES pH 7.4, 1 mM MgCl_2_, 100 mM KCl, 0.1 mg/ml BSA and 1 mM DTT), aliquots of the immunocomplexes on beads were incubated with GTP[α-^32^P]-loaded GST-Rheb (1 μg/per reaction) at 30 °C with agitation. To stop the reactions at the indicated times, we added a 2 volume of Rheb wash buffer including 1 mg/ml BSA. For isotope-based Rheb-GAP assay, [α-^32^P]-GTP and -GDP were purified from supernatant with 20 μl Rheb elution buffer (0.5 mM GDP, 0.5 mM GTP, 5 mM DTT, 5 mM EDTA, and 0.2% SDS) at 68 °C for 20 min, and then resolved by thin layer chromatography (TLC) on polyethyleneimine (PEI) cellulose with 0.75 M KH_2_PO_4_. Relative [α-^32^P] GTP and GDP levels were quantified using ImageJ software (Wayne Rasband, NIH).

## Data availability

Data sharing not applicable to this article as no datasets were generated or analyzed during the current study.

## Supporting information

This article contains supporting information.

## Conflict of interest

The authors declare that they have no conflicts of interest with the contents of this article.
