# Supplementary material for: CBAP regulates the function of Akt-associated TSC protein complexes to modulate mTORC1 signaling
Source: J Biol Chem. 2023 Nov 8;299(12):105455. doi: 10.1016/j.jbc.2023.105455 (PMC10698277; doi:10.1016/j.jbc.2023.105455)
Supplement: Supplemental Figs. S1–S5 [file mmc1.pdf]

Supplementary Figure 1

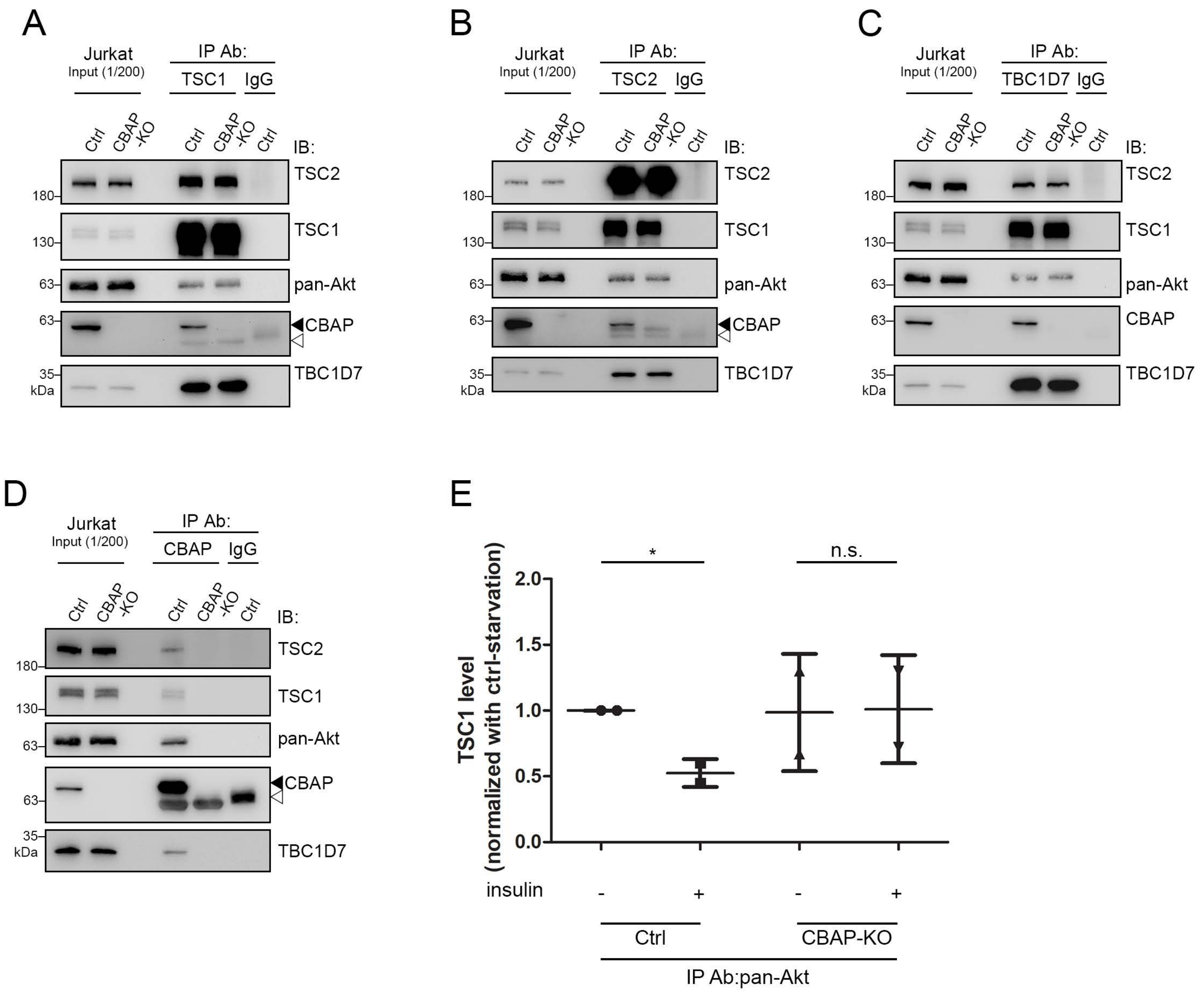

Supplementary Figure 1. Interaction among endogenous TSC1, TSC2, TBC1D7, At and CBAP in Jurkat cells.

Immunoprecipitation was performed using lysates from Jurkat leukemia cells with antibodies against TSC1 (A), TSC2 (B), TBC1D7 (C), and CBAP (D), followed by immunoblotting with the indicated antibodies. (E) The signal intensity of TSC1 was quantified and normalized with the value in ctrl-starvation samples. Statistical significance was calculated using the Student's t-test, where * indicate P < 0.05.

Supplementary Figure 2

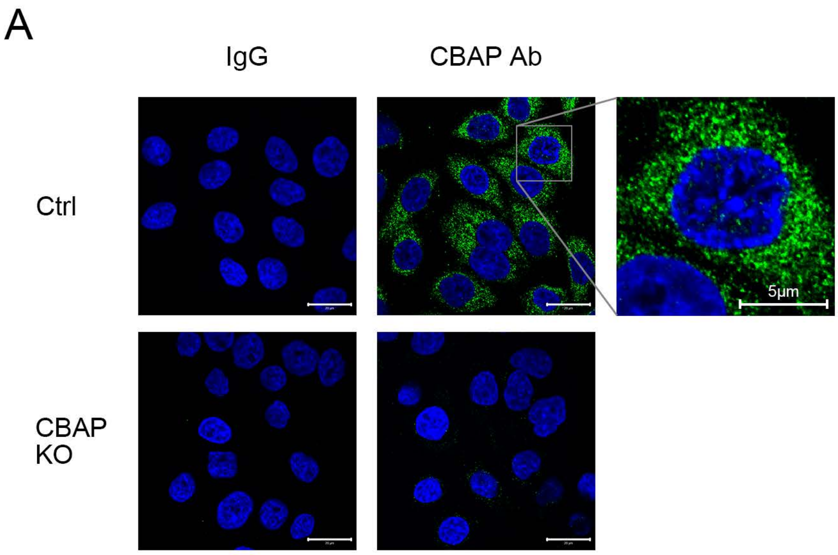

Supplementary Figure 2. Immunofluorescence staining of CBAP in control and CBAP-knockout HeLa cells.

Ctrl or CBAP-KO HeLa cells were fixed and incubated with a mouse anti-human CBAP monoclonal antibody as described in reference 32. Scale bars, 20µm.

Supplementary Figure 3

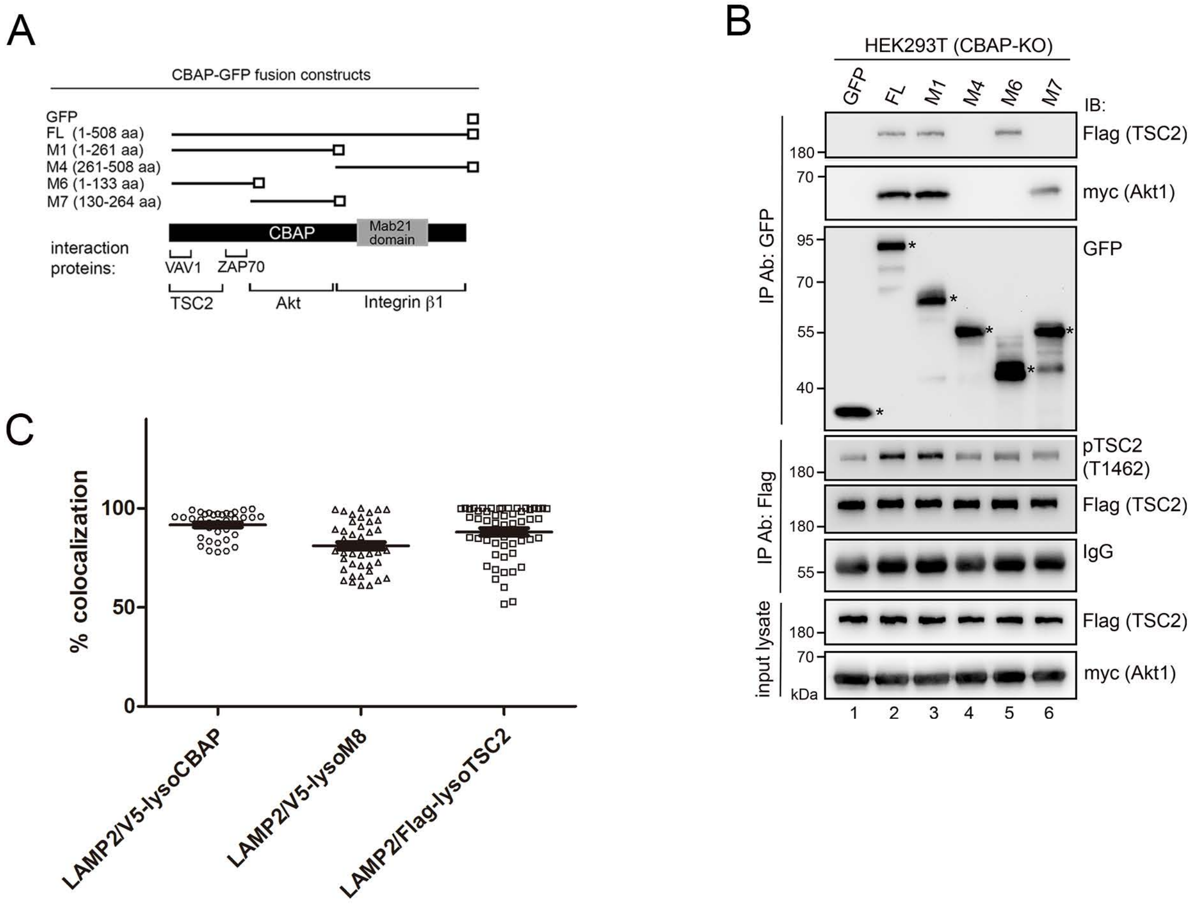

Supplementary Figure 3. CBAP interacts with Akt and TSC2 via distinct region
(A) Schematic representation of different truncated CBAP-GFP constructs and the domains of CBAP that interact with Akt and TSC2. (B) Akt-mediated phosphorylation of TSC2 requires the simultaneous interaction of CBAP with Akt and TSC2. Myc-Akt1 and Flag-TSC2 were co-transfected into CBAP-KO HEK293T cells with the truncated CBAP-GFP constructs. We immunoprecipitated with either anti-GFP or anti-Flag Ab and blotted the immunoprecipitates with the indicated antibodies. (C) Expression of p18-tagged CBAP, M8, or TSC2 was highly enriched on lysosomes. To show this, HeLa cells were first transfected with Lyso-CBAP, Lyso-M8, or Lyso-TSC2 before immunofluorescence staining with the indicated anti-tag and anti-Lamp2 antibodies. Percentage of colocalization was calculated by measuring the Pearson correlation coefficient for over 30 cells selected from 5 to 10 representative confocal images in a triplicate experiment.

Supplementary Figure 4

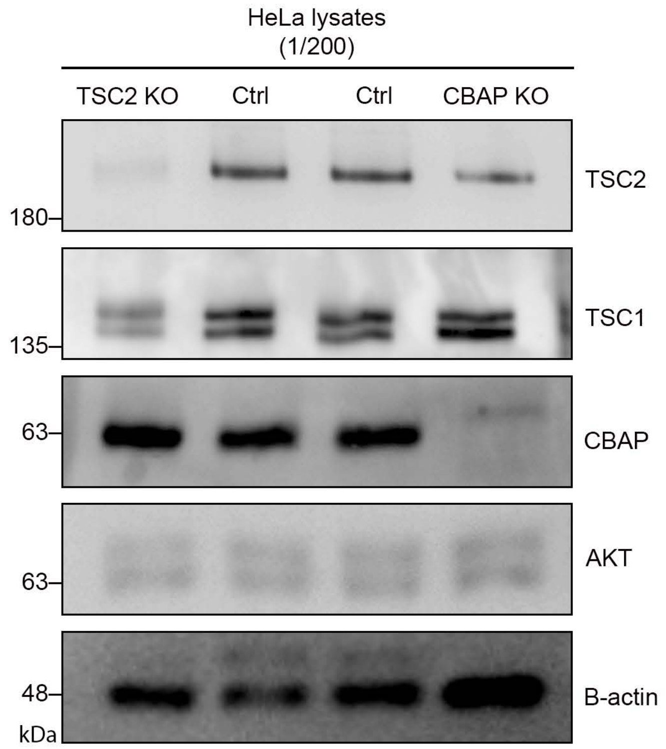

Supplementary Figure 4. Loading control of HeLa cell lysates used for isotope GAP activity assay.
Equal amount of HeLa cell lysates from TSC2-KO, ctrl and CBAP-KO, subjected to immunoblotting with the indicated antibodies.

Supplementary Figure 5

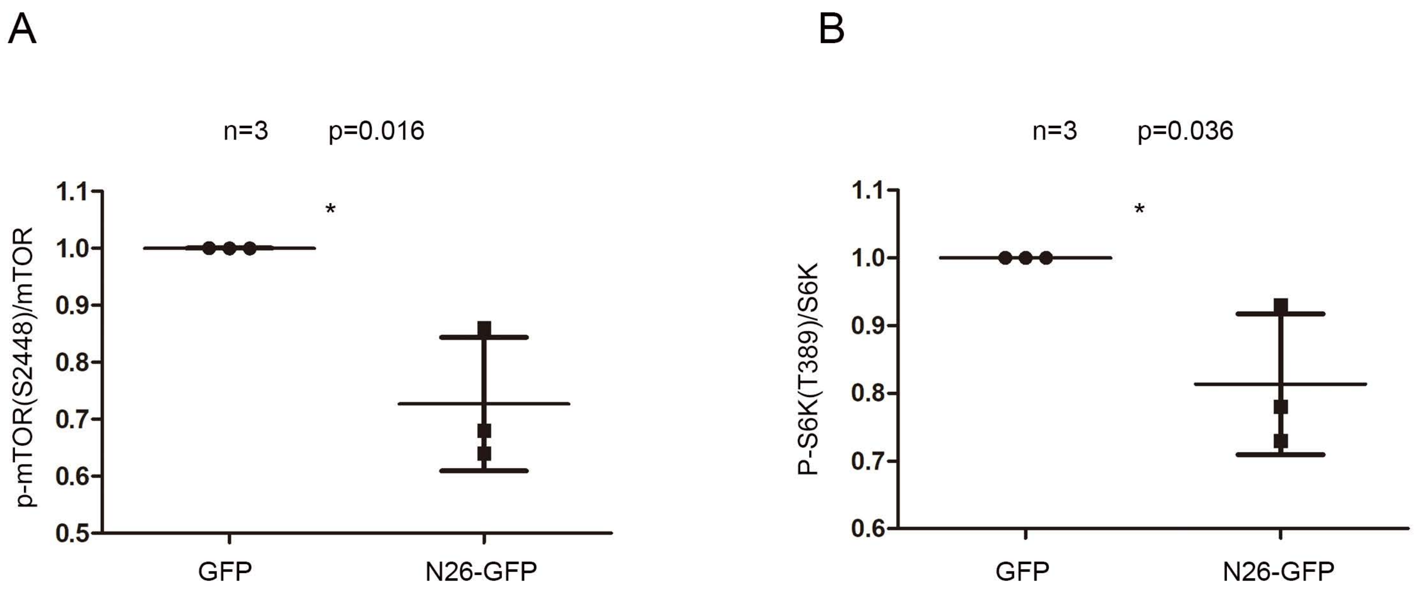

Supplementary Figure 5. Expression of N26-GFP decreases the phosphorylation of mTOR and S6K in Jurkat cells.

The signal intensity of p-mTOR and mTOR, as well as p-S6K and S6K in N26-CBAP-expressing cells (Fig. 6A) was quantified, and the values of p-mTOR/mTOR (A) and p-S6K/S6K (B) were normalized to those in the GFP-transfected control sample. Statistical significance was calculated using the Student's t-test, with *, $P < 0.05$.
